# Comment on Grivas et al. Morphology, Development and Deformation of the Spine in Mild and Moderate Scoliosis: Are Changes in the Spine Primary or Secondary? *J. Clin. Med.* 2021, *10*, 5901

**DOI:** 10.3390/jcm11051160

**Published:** 2022-02-22

**Authors:** Steven de Reuver, Tom P. C. Schlösser, Moyo C. Kruyt, Keita Ito, René M. Castelein

**Affiliations:** 1 Department of Orthopaedic Surgery, University Medical Center Utrecht, 3584 CX Utrecht, The Netherlands; s.dereuver-4@umcutrecht.nl (S.d.R.); t.p.c.schlosser@umcutrecht.nl (T.P.C.S.); m.c.kruyt@umcutrecht.nl (M.C.K.); k.ito@tue.nl (K.I.); 2 Department of Biomedical Engineering, Eindhoven University of Technology, 5600 MB Eindhoven, The Netherlands

With great interest, we have read the article entitled “Morphology, Development and Deformation of the Spine in Mild and Moderate Scoliosis: Are Changes in the Spine Pri-mary or Secondary?” by Grivas et al., an incredibly interesting read [1]. This study covers the important but divided topic of sagittal spinal alignment as a causal factor in the etiology of idiopathic scoliosis, and we would like to compliment the authors on their work. The purpose was to study “*the sagittal profile of the onset and mild idiopathic scoliosis, using the radiography, the surface topography and the scoliometer readings of children with idiopathic scoliosis*”. The data presented in this study contribute to the discussion; however, we believe it should be interpreted with caution since there are substantial methodological limitations, and these results should not seek to provide a definitive answer.

1. The authors try to translate their data towards a definitive answer on whether sagittal spinal alignment is a primary etiological factor of idiopathic scoliosis. In contrast to the defined aim, they studied the sagittal profile of patients with already established idiopathic scoliosis (mean Cobb angle of 28 and 30 degrees) cross-sectionally and without radiographic sagittal measurements. It has been widely described that the apical deformation in idiopathic scoliosis is characterized by apical lordosis and alters the regional and global sagittal profile [2]. Additionally, the cross-sectional nature of these data can only suggest a relationship; the only way to discriminate between cause and effect would be a prospective longitudinal study.
2. The authors summarize the theoretical background and state that the “*lateral spinal profile*” was commonly considered to be a primary etiological factor of idiopathic scoliosis, because the thoracic kyphosis apex is located in a higher thoracic vertebra (therefore, more vertebrae are posteriorly inclined), which creates conditions of greater rotational instability and, therefore, increases vulnerability for idiopathic scoliosis development. We appreciate this simplification; however, it misses an important nuance: A longer posteriorly inclined segment was previously shown to be associated with thoracic scoliosis, but not necessarily with (thoraco) lumbar scoliosis. For the latter, in fact, a shorter but more steep posteriorly inclined segment with a more distal kyphotic apex may be much more relevant [3,4]. From the scoliosis patients included in the study, 9 out of 17 had a primary (thoraco) lumbar curve, and the primary thoracic curves were not analyzed separately. This actually counterbalances the outcome parameter of this study, since only for thoracic scoliosis, this apex is expected to be higher.
3. The efforts of the authors to provide a sample-size calculation is appreciated, and since the calculated *n* = 17 for the cases (total of thoracic and (thoraco) lumbar curves) are precisely met, they are confident that the non-significant difference of 0.39 ± 0.11 in scoliosis vs. 0.44 ± 0.08 in controls (*p* = 0.134) is not a type II error. The type of power analysis and whether the test was one or two sided is not mentioned. The 0.l standard deviation seems to have been well chosen looking at the results; however, the rationale for a 0.1 margin was not given. A 0.1 margin for equivalence seems rather broad, since in the controls, the mean difference between boys and girls is only 0.02, and it is likely that slight, not drastic, inter-individual sagittal spinal alignment variability predisposes for scoliosis development. If this study used a smaller margin, this would sharply increase the required sample size [5].
4. There is a fair chance of selection bias as only two of the 17 scoliosis patients were boys (which is expected given the epidemiology of idiopathic scoliosis), while the sex distribution of the controls was more even with 15 girls and 11 boys. Moreover, the age distribution of the controls and scoliosis series does not match, and they include juveniles as well as adolescents. The mean VP-KA/VP-LA ratio in the controls was higher in girls, representing a relatively lower kyphotic apex than boys, which indicates a sex difference. Therefore, the scoliosis group, which mostly consisted of girls, is biased to have a higher ratio. Interestingly, the ratio was still lower in the scoliosis group compared to controls, although not significant. This is important, since a trend towards a smaller VP-KA/VP-LA ratio (i.e., higher kyphotic apex) in idiopathic scoliosis, probably corresponds to a relatively longer posteriorly inclined segment, which is opposite to the authors’ conclusion but in line with other hypotheses in the literature [3].
5. In addition to comparing the two groups, the authors demonstrate that the VP-KA/VP-LA ratio did not significantly correlate with the scoliometer trunk asymmetry measurements (r = 0.211, *p* = 0.416). Therefore, the authors conclude that the hypothesis of a larger posteriorly inclined spinal segment being a primary etiological factor for idiopathic scoliosis is not confirmed. This study did not include a power analysis for this specific test, possibly introducing a type II error. However, most importantly, the correlation with curve severity is not relevant for the sagittal spinal alignment, since it has been hypothesized to influence scoliosis risk, not severity. Scoliosis severity is probably mostly correlated with time from onset, not the sagittal profile.
6. Throughout the manuscript, the authors interchange the terms ‘posteriorly inclined segment’ and ‘hypokyphosis’; however, we would like to emphasize that these are distinct. Based on previous findings and the results presented, we agree that hypokyphosis is not likely the primary causal factor in idiopathic scoliosis, rather a passive result of the combined effect of the rotation and anterior opening of the apical intervertebral disc spaces [6]. However, the individual posteriorly inclined spine, specifically the relative length and inclination of that segment, is hypothesized to be a risk factor for scoliosis development, which is supported by recently published prospective data [4].

In conclusion, the statistical limitations, interchange of distinct terminology and the cross-sectional nature of this study, in our mind, may lead to a less conclusive statement that sagittal alignment has no place in idiopathic scoliosis etiology. If anything, we feel that the hypothesis rejected by the authors, that the posteriorly inclined segment is larger in scoliosis patients, is actually suggested in their data but masked by poor group selection of both the scoliosis and the control group. Regardless of the shortcomings related to these types of studies, we commend the authors for their efforts in exploring the sagittal spinal alignment in relation to idiopathic scoliosis etiology, and we look forward to their future contributions.
